# Efficacy of CMX001 as a Post Exposure Antiviral in New Zealand White Rabbits Infected with Rabbitpox Virus, a Model for Orthopoxvirus Infections of Humans

**DOI:** 10.3390/v3010047

**Published:** 2011-01-24

**Authors:** Amanda D. Rice, Mathew M. Adams, Greg Wallace, Andrew M. Burrage, Scott F. Lindsey, Andrew J. Smith, Daniele Swetnam, Brandi R. Manning, Stacey A. Gray, Bernhard Lampert, Scott Foster, Randall Lanier, Alice Robertson, George Painter, Richard W. Moyer

**Affiliations:** 1 Department of Molecular Genetics and Microbiology, University of Florida, 1600 SW Archer Rd, Gainesville, FL 32610, USA; E-Mails: amandar@ufl.edu (A.D.R.); mathew.adams@csiro.au (M.M.A.); wallace.gregt@gmail.com (G.W.); andrew91386@gmail.com (A.M.B.); slindsey@med.miami.edu (S.F.L.); andyjsmith84@gmail.com (A.J.S.); dmswetnam@gmail.com (D.S.); brmanning10@gmail.com (B.R.M.); gray.staceya@gmail.com (S.A.G.); 2 Chimerix, Inc., 2505 Meridian Parkway Suite, 340 Durham, NC 27713, USA; E-Mails: blampert@chimerix.com (B.L.); sfoster@chimerix.com (S.F.); rlanier@chimerix.com (R.L.); arobertson@chimerix.com (A.R.); gpainter@chimerix.com (G.P.)

**Keywords:** poxvirus, antiviral, CMX001, rabbitpox, smallpox treatment

## Abstract

CMX001, a lipophilic nucleotide analog formed by covalently linking 3-(hexdecyloxy)propan-1-ol to cidofovir (CDV), is being developed as a treatment for smallpox. In the absence of human cases of smallpox, new treatments must be tested for efficacy in animal models. Previously, we demonstrated the efficacy of CMX001 in protecting New Zealand White rabbits from mortality following intradermal infection with rabbitpox virus as a model for smallpox, monkeypox and for treatment of adverse reactions to smallpox vaccination. Here we extend these studies by exploring different dosing regimens and performing randomized, blinded, placebo-controlled studies. In addition, because rabbitpox virus can be transmitted via naturally generated aerosols (animal to animal transmission), we report on studies to test the efficacy of CMX001 in protecting rabbits from lethal rabbitpox virus disease when infection occurs by animal to animal transmission. In all cases, CMX001 treatment was initiated at the onset of observable lesions in the ears to model the use of CMX001 as a treatment for symptomatic smallpox. The results demonstrate that CMX001 is an effective treatment for symptomatic rabbitpox virus infection. The rabbitpox model has key similarities to human smallpox including an incubation period, generalized systemic disease, the occurrence of lesions which may be used as a trigger for initiating therapy, and natural animal to animal spread, making it an appropriate model.

## 1. Introduction

CMX001 is a lipophilic nucleotide analog formed by covalently linking 3-(hexdecyloxy)propan-1-ol to cidofovir (CDV). Cidofovir (marketed as Vistide^®^), an antiviral drug approved for the treatment of CMV-retinitis [1,2], is an alternative substrate inhibitor of the DNA polymerases encoded by orthopoxviruses, and has shown activity in lethal models of poxvirus infection using mice and monkeys [3–7]. Although CDV is the only antiviral drug currently available for use in the event of a smallpox outbreak, its utility in an attack would be limited since it must be administered by slow intravenous infusion and has the potential for significant nephrotoxicity [8,9]; currently there is no approved antiviral for treatment of smallpox. In an effort to address the need for an orally available antiviral drug for human poxvirus infections, a lipid conjugate of CDV was synthesized by covalently coupling CDV to hexadecylpropanediol; the resulting compound is referred to as CMX001 hereforth. The conjugate was designed to resemble a natural phospholipid and utilize natural uptake pathways to achieve oral availability, high uptake in target cells, and overall improved Absorption, Distribution, Metabolism, Excretion (ADME) profiles [10]. Because variola virus has been eliminated from the environment, no new cases of smallpox have been reported since 1977 [11] and therefore it is not possible to test the efficacy of potential treatments in human trials with smallpox. Rabbitpox virus infection of rabbits has been used as a model for smallpox as well as general orthopoxvirus infections of humans, such as monkeypox virus infections, since its discovery in the 1930s [12–16].

We have used intradermal inoculation and natural aerosol (animal to animal) transmission to infect New Zealand White (NZW) rabbits with rabbitpox virus as models to evaluate the efficacy of CMX001 as a potential treatment for orthopoxvirus infection. Here we examine the effectiveness of CMX001 administered after virus exposure and in rabbits infected from index animals. Our results demonstrate that CMX001 is effective in preventing mortality and reducing morbidity when dosed after the onset of acute symptoms as measured by the presence of secondary lesions.

## 2. Results and Discussion

### 2.1. Regimen of CMX001 Required for Protection from Rabbitpox virus (RPV) Disease and Death

*In vitro* studies with human PBMCs showed that the half life of the active metabolite of CMX001 was up to 6.5 days [17] and *in vitro* data indicates that the active antiviral in common with CMX001 and cidofovir (cidofovir diphosphate) has a long intracellular half life [18]. Therefore, it was hypothesized that a single treatment with CMX001 might be sufficient to prevent lethal RPV disease as described in the accompanying paper [19]. We tested the efficacy of CMX001 in intradermally infected rabbits when dosed one, two, or three times over the course of five days starting on 3 or 4 dpi (ear lesions typically occur beginning on 4 dpi). As shown in Table 1, all groups in which treatment was begun at 3 dpi survived RPV infection. The survival rate of animals beginning treatment at 4 dpi was 66% regardless of whether one, two or three doses of CMX001 were administered. The animal euthanized from the 4 dpi treatment group was due to severe respiratory disease, the animal euthanized from the 4/6 (4, 6 dpi) treatment group was due to weight loss and the animal from 4/6/8 (4, 6, 8 dpi) treatment group was removed due to severe respiratory distress. Thus it appeared that one to three doses of CMX001 were sufficient to provide a survival benefit. In fact, all groups receiving CMX001 demonstrated a significant survival benefit when compared to the pooled vehicle controls across different experiments (14/14 mortalities) (data not shown).

The animals treated with CMX001, regardless of the number of doses or day treatment was initiated, exhibited less weight loss than the vehicle treated animals (Figure 1A and D). Both 3/5/7 (3, 5, 7 dpi treated) and 4/6/8 treatment groups exhibited less weight loss and a return to weight gain more quickly than the single or double dosed treatment groups. There was little difference in weight change profiles between 3 and 3/5 (3, 5 dpi treated) and 4 or 4/6 (4, 6 dpi) treatment groups.

All groups exhibited temperature spikes during the course of the experiment. Animals that received CMX001 once or twice exhibited a fever from 5 to 11 dpi while animals that received CMX001 three times exhibited a fever from 5 to 9 dpi with a lower overall maximum temperature (Figure 1B and E). Generally, the more times the rabbits were given CMX001, the less severe their temperature spikes were. The overall disease as measured by clinical scores over the course of the experiment showed a decrease in disease severity of all treatment groups as compared to vehicle treated animals (Figure 1C and F). There was, however, little difference in clinical score profiles over the course of the experiment between any of the treatment groups when compared to number of doses of CMX001 received.

The primary lesion in 3 and 4 dpi treatment groups was fairly large, covering a large area of the animals’ flank and showed a good deal of necrosis. Animals in these treatment groups also had numerous widespread secondary lesions localized mainly in the eyes, ears, and mouth. The animals from treatment groups 3/5 and 4/6 dpi exhibited smaller primary lesion sites and less severe necrosis with a moderate number of secondary lesions. Treatment groups 3/5/7 and 4/6/8 dpi exhibited fairly mild primary lesions characterized by less necrosis and less edema and swelling (Figure 1G) and had fewer secondary lesions, all localized mainly in the eyes, ears, and mouth. The primary lesions are characterized not only by the highly visible area of necrosis but also by the area of edema which defines the overall lesion size. The area of edema does not produce a swelling of the flank but rather a thickening of the skin up to 3 cm in depth and the photographs do not show this clearly.

Using the maximum clinical score and weight loss as indications of maximal illness, it was observed that treatment with CMX001 provides a reduction in the disease severity for all treatment groups (Figure 2A). Animals that began treatment on 3 dpi, regardless of number of doses they received, exhibited a lower maximal clinical score over the course of the experiment suggesting the number of doses may not have an immense impact on outcome when initiated early in the disease. Similarly, among the animals treated starting at day 4, there was little difference in disease severity regardless of the number of doses the animals received. The maximum weight loss over the course of the experiment paralleled the maximal clinical score (Figure 2B). All animals that received treatment exhibited a less severe weight loss maximum as compared to the vehicle treated animals and, in general, animals that began treatment at 4 dpi, regardless of the number of doses, exhibited less weight loss.

### 2.2. Randomized, Blinded, Placebo Controlled Studies

To unequivocally demonstrate the efficacy of CMX001, we performed randomized, blinded, placebo-controlled studies that were appropriately powered to achieve statistical significance. In this series of experiments, rabbits were intradermally inoculated with RPV and at the first sign of secondary lesions in the ears, CMX001 treatment was initiated for each rabbit on an individual basis. Both males and females were used to investigate the possibility of gender bias in this experiment as previous studies utilized only female rabbits. Rabbits were randomly assigned to treatment groups and blinded dosing formulations containing either CMX001 or vehicle were prepared by Chimerix and shipped to the University of Florida for testing. Due to limitations of study size, three separate studies (of 12 CMX001 treated and 12 control animals) were conducted. Within each study, all rabbits received either one, two or three doses of blinded treatment, thus the number of doses was not blinded.

There was a statistically significant increase in survival rates when CMX001 was administered to RPV infected rabbits as compared to vehicle treated age and sex matched control animals (Table 2) using Fisher’s exact test. A single dose of CMX001 protected seven of 12 animals while two doses protected eight of 12. For animals that received three doses of CMX001, 11 of 12 survived. In contrast, only four of 36 animals receiving vehicle only survived. There was no large difference in the day of death for any of the animals that were euthanized due to severe RPV induced disease.

All treatment groups exhibited weight loss over the course of the experiment; however the groups receiving two and three doses of CMX001 exhibited less weight loss as compared to the group receiving one dose of CMX001 and the vehicle treated group (Figure 3A). The temperature profiles for the groups were indistinguishable from that of the vehicle treated animals with all animals exhibiting fevers from 3 to 8 dpi (Figure 3B). There was a general trend for animals receiving a single dose of CMX001 to exhibit a higher temperature; this difference however was not statistically significant. There was a difference in clinical score profiles for the course of the experiment with the 3 dose group exhibiting a lower clinical score consistently (Figure 3C). Primary lesions in treated animals began to heal and decrease in size after the initiation of CMX001 treatment. The primary lesions are shown in Figure 6D at 8 dpi showing little difference between one and two doses of CMX001 as compared to three doses in which the primary lesions rapidly decreased in swelling and began scabbing.

Maximal illness over the course of the experiment was analyzed by the average maximum clinical score, weight change and primary lesion size. All animals treated with CMX001 exhibited a statistically significant decrease in the maximum clinical score as compared to vehicle treated animals (Figure 4A). Within the treatment groups there was a general trend observed in which the more doses of CMX001 received, the less severe disease. The maximum average weight change again demonstrated a similar trend to that of clinical score in which the more doses of CMX001 animals received, the lower the weight loss observed (Figure 4B). There was a statistical decrease in the weight loss of two and three doses as compared to that of vehicle treated animals. There was a decrease in average maximum size of the primary lesion as compared to vehicle treated animals for all CMX001 treated groups (Figure 4C). No differences in the maximum lesion size among the CMX001 treatment groups were observed. The level of virus dissemination was evaluated in this experiment; however the results were uninformative most likely due to limitations of live virus detection methods (data not shown).

### 2.3. Rabbitpox Infection via Natural Aerosol Model Review

In addition to the ID route, lethal infection in the rabbit/RPV model can also occur by animal to animal aerosol transmission. In this natural aerosol model virus transmission occurs from index (1000 pfu RPV intradermally infected) to sentinel (uninfected) animals. Transmission has been observed when sentinel animals are either co-housed or in adjacent cages to the index animals [12] and is believed to be very similar to the transmission that occurs in smallpox in humans. While it is difficult to determine the infectious dose in this natural animal to animal aerosol transmission model it is believed to be very low. This model utilizes the natural conditions under which RPV has been shown to transmit virus throughout rabbit colonies such as in Utrecht and Rockefeller University. The disease timeline is shown in Figure 5 for the sentinel co-housed animals.

While the amount of virus transmitted to the sentinel animals is not readily measured or controlled, the timing of sentinel animal infection can be inferred by a reverse tracing of clinical symptoms. Although it is possible that some rabbits may become inoculated via routes other than the respiratory tract, we have examined this issue and have never found any evidence of primary skin lesions on infected sentinel animals nor were we able to induce infections of sentinel through waste material derived from infected sentinel animals [20]. Indeed, the only difference clinically between infected index (intradermally infected) and sentinel (animal to animal natural aerosol infected) animals is a neck swelling of sentinel animals (Figure 7D) which is similar to what we observe in intranasally infected rabbits [21] and consistent with an aerosol inoculation from index animals [13].

There is a 23.1% survival rate of natural aerosol infected animals (Figure 6A). The difference in survival rates as compared to the highly lethal intradermal infected rabbits is perhaps due to dose; 10 to 100 times more RPV is required to cause lethal disease when virus is administered intranasally [21]. The first clinical signs of systemic disease following animal to animal transmission are the presentation of a fever (Figure 6C) and failure to gain weight (Figure 6B) that begin 9–10 dpi. Weight loss is approximately 10% from the maximum weight during the course of the experiment and occurs at 13 dpi. The severity of fever observed following animal to animal transmission is 40.6 °C ± 0.72 on 14 dpi and not statistically or biologically different from that observed with intradermally infected animals with a maximum temperature of 40.8 °C ± 0.34 on 5 dpi. The severity of disease is quantified using the clinical score (Figure 6D); RPV infected animals exhibit severe disease with an average maximum clinical score of 13.89 ± 1.43 points out of a possible 25 points (note that the natural aerosol infected animals have a lower maximum score because no primary lesion is present).

Secondary lesions are observed in the ears first and generally appear 12–24 hours after the first sign of a fever is observed, following the same disease progression as intradermally infected animals. The lesions first appear in the ears as small red spots typically found at the bend of a blood vessel and are easily visualized by backlighting the ears. The progression of these small red spots to pustular lesions on the ears is shown in Figure 7A. We have noted that the ear lesions are larger and more pronounced in aerosol infected animals. Secondary lesions are also found on the nose, eyelids (Figure 7B), in and around the mouth, genitals and as a rash across the body of the rabbit.

Animals infected with RPV are euthanized due to respiratory distress characterized by open mouth breathing, a decrease in respiration rate to below 40 breaths per minute or severe lung sounds. The respiratory symptoms begin at approximately 10 dpi and progress to euthanasia criteria levels by 12–17 dpi. At the time of euthanasia it is typical to observe profuse mucopurulent discharge from the nostrils (Figure 7C) and severe discharge from the eyes.

RPV in rabbits share many features of variola in humans. In addition to the fact that replication and spread of RPV and variola within the host are believed to be very similar [11] and both viruses spread by natural aerosol transmission [22–25]. While there are some differences between RPV via natural aerosol transmission to variola, including a shortened incubation period, both viruses produce a systemic viremia and characteristic dermal rash. The natural aerosol transmission of RPV is an important model for evaluating both antivirals and vaccines as it is the closest model to human exposure to smallpox currently available.

### 2.4. Treatment of Natural Aerosol Infected Animals

The efficacy of CMX001 in rabbits infected with RPV by natural aerosol was investigated. Sentinel animals were co-housed with index animals intradermally inoculated with 1000 PFU of RPV. Sentinel animals develop ear lesions and an elevated temperature at 8–10 days after infection of the index animal, suggesting sentinels received virus from the intradermally infected index animals at about 4–6 days after the initial intradermal infection. The sentinel rabbits were then observed for the development of secondary lesions as a trigger to initiate CMX001 treatment. Animals were given a total of one, two or three doses of CMX001 (20 mg/kg per dose) spaced every other day beginning on the day secondary lesions were detected in the ears of the animals. Secondary lesions appeared in the sentinel animals between seven and nine days post infection of the index animals. No vehicle-treated animals survived, however two/three of the animals in each CMX001 treatment group survived (Table 3).

Overall, the CMX001 treatment groups exhibited less severe disease as compared to vehicle treated animals. CMX001 treated animals exhibited a trend of less weight loss than vehicle treated animals (Figure 8A). All animals exhibited a fever (body temperature of 39.5 °C or higher), however animals receiving CMX001 tended to have lower average maximum temperatures as compared to vehicle treated animals (Figure 8B). All animals developed RPV disease symptoms as shown by the profiles of clinical scores for each group (Figure 8C). Again, as observed with weight loss and degree of fevers observed, animals receiving CMX001 tended to show less severe disease and did not develop the disease on average as quickly as the vehicle treated animals. While the number of animals in this study was small, making statistical significance difficult to achieve, the data obtained suggest that CMX001 is effective in treating rabbits infected with RPV via the aerosol route and therefore supports the use of CMX001 against smallpox in humans where exposure is via aerosol.

## 3. Experimental Section

### 3.1. Cell and Virus Growth

CV-1 cells were maintained in Minimum Essential Media (MEM) with Earle’s Salts (Gibco, New York, USA) supplemented with 2 mM glutamine (Media Tech, Virginia, USA), 50 U/mL penicillin G and 50 μg/mL streptomycin (Media Tech), 1 mM sodium pyruvate (Media Tech), and 0.1 mM nonessential amino acids (Media Tech), and 10% v/v FBS (Gibco).

Rabbitpox virus (RPV) was obtained from ATCC and virus stocks used in these experiments were ≤5 passages from the original stock and have maintained virulence as evidenced by an LD50 in 9-week old NZW rabbits of approximately 10 PFU by the intradermal route. All viruses for animal infections were pad purified over 36% sucrose using standard methods and resuspended in PBS. Viruses were tittered on CV-1 cells using standard methods. [26,27]

### 3.2. Housing of Animals

For intradermally infected rabbits, 8 week (3–4 lb) old New Zealand White rabbits were obtained from Myrtle’s Rabbitry (Thompson Station, Tennessee, USA) and housed in standard stainless steel solid back and side cages at 20 °C and 12 h light / 12 h dark regime. All animals obtained a unique ear tattoo number in the left ear prior to arrival at the study site. Animals were allowed to acclimate to their surroundings for 5–10 days prior to RPV infection. Food and water were available to the animals *ad libitum*. Rabbits received apples, alfalfa cubes, or fresh greens daily for enrichment.

For animal to animal natural aerosol transmission studies, six rabbits were co-housed in a 160 × 80 cm cage with shredded paper bedding and fed *ad libitum*. Two rabbits were infected intradermally with RPV as described previously (index animals) and housed with four uninfected rabbits (sentinel animals). Upon onset of severe respiratory disease, the index animal was euthanized. At the appearance of secondary lesions the sentinel animals were transferred to a standard steel single rabbit housing cage for the remainder of the experiment.

All animal procedures were approved by the University of Florida Institutional Animal Care and Use Committee.

### 3.3. Animal Infections

Intradermal infection was performed by bilateral shaving of both thighs of the rabbit, sterilizing with an isopropanol wipe followed by intradermal injection of 100 or 1000 pfu RPV using a 27 gauge needle [12]. The injection site was then traced using a black permanent marker to note the site of infection.

### 3.4. Monitoring of Animals

Rabbit were microchipped at the time of infection to transmit body temperature and unique identification number (Bio Medic Data Systems, Seaford, DE). Rabbits underwent a complete physical daily. Rabbits were euthanized upon the onset of severe respiratory distress (labored, extremely slow or open mouth breathing) or weight loss of greater than 15% of initial body weight.

Clinical scores for each rabbit each day were generated by assigning numerical values of 0 to 4 for all clinical measurements obtained during the course of the evaluation of each rabbit. These measurements included: Weight change (loss, no change or gain); body temperature (low, within normal limits, fever); respiration rate (normal, depressed); heart rate (normal, decreased); intake and output; overall attitude and posture; presence of secondary lesions (number of secondary lesions and number of sites present); and primary site of infection condition (degree of reaction). These scores are used as a measure of disease severity where the higher the number the more severe the disease.

Weight change was calculated as the percent change from weight on the day of infection with RPV. All statistical analysis is performed using an unpaired t test for each group measurement as compared to vehicle treatment groups.

All animal procedures and euthanasia were carried out according to the University of Florida IACUC guidelines.

### 3.5. CMX001 Dosing of Animals

Animals were trained for 4–5 days prior to the initiation of the study to readily accept the 10% sucrose solution used to deliver CMX001 (HDP-CDV). The 10% sucrose training solution, vehicle solution and CMX001 was administered orally using a 1 ml disposable syringe with the end dipped in granulated sugar and fed orally to the animals. The total volume of liquid the animals received was approximately 1.0 mL. Dry powdered CMX001 (Chimerix Inc, North Carolina, USA) was dissolved in 10% sucrose in water with food coloring to the required concentration. Vehicle treated animals received 10% sucrose solution only. In a previous pharmacokinetic study, all rabbits treated with CMX001 for seven days at doses up to 25mg/kg/day survived to the scheduled termination. In this special issue there is a review on the development of CMX001 for further toxicological data.

## 4. Conclusions

Previously, we demonstrated the efficacy of CMX001 in protecting New Zealand White rabbits from mortality following intradermal infection with rabbitpox virus as a model for smallpox. Here we extended these studies by exploring different dosing regimens and performing randomized, blinded, placebo-controlled studies. Lesions in the ears are the result of systemic viral spread within the animal and the first appearance of ear lesions was used in these studies as a trigger to initiate treatment to model how smallpox might be recognized and treated in the event of a release. CMX001 was administered orally as a single dose of 20 mg/kg, two doses of 20 mg/kg (one dose on the day of lesions and a second dose two days later) or three doses of 20 mg/kg (one dose on the day of lesions with additional doses two and four days later). In randomized, blinded, placebo-controlled studies, treatment of intradermally infected rabbits with CMX001 at the first appearance of ear lesions provided a significant survival benefit compared to vehicle treated animals. This was true for each CMX001 treatment group regardless of the number of doses. The need for relatively few doses of CMX001 is most likely explained by the long half-life of the active antiviral species, cidofovir diphosphate, in cells. After entering the cell, the lipid moiety of CMX001 is removed by cellular phospholipases to release free cidofovir which is then anabolized to the active antiviral form, cidofovir diphosphate. Cidofovir diphosphate is a nucleoside triphosphate mimic and is incorporated by the viral DNA polymerase. Despite the efficacy of the single dose treatment, the three dose treatment appeared to result in an improved survival rate and reduced disease severity.

We also explored the efficacy of CMX001 treatment in animals infected via a natural aerosol, which provides an animal to animal transmission model of orthopoxvirus infections. In this model, index animals were intradermally inoculated and then co-housed with sentinel animals to allow for transmission of the infection to the uninfected animals. The sentinel animals were then treated with CMX001 at the first signs of lesions in the ears. As with the intradermal infection model, one, two, or three doses of CMX001 appeared to significantly reduce disease symptoms and have a survival benefit.

Overall, the results presented demonstrate that CMX001 is an effective treatment for symptomatic rabbitpox virus infection in rabbits. Attributes of the rabbitpox model including an incubation period, generalized systemic disease, the occurrence of lesions which may be used as a trigger for initiating therapy, and natural animal to animal spread make it a particularly relevant model for smallpox in humans. The delay of treatment initiation and dosage flexibility demonstrate the feasibility of CMX001 for use in treatment of any orthopox virus infections in humans.

## Figures and Tables

**Figure 1:**
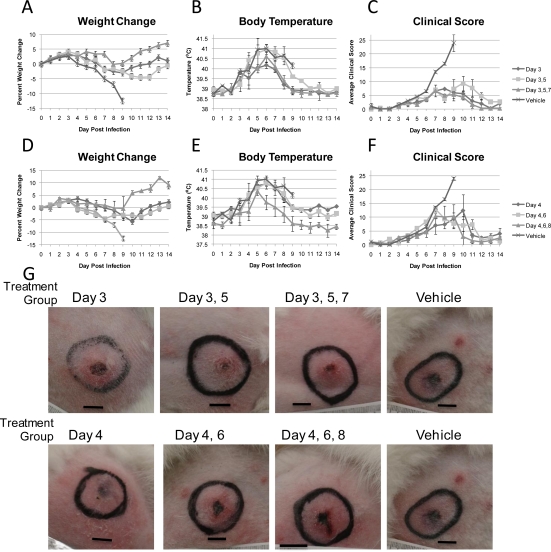
Clinical observations for evaluation of 1, 2 or 3 doses of CMX001 given every other day beginning at day 3 or 4 post infection. Animals were dosed at concentrations and schedules as outlined in Table 1. **(A)** Average weight change from weight at day of infection for animals that began treatment on day 3 post infection. Negative values indicated weight loss. **(B)** Average body temperatures for animals that began treatment on day 3 post infection. **(C)** Average clinical scores for animals that began treatment on day 3 post infection. **(D)** Average weight change from weight at day of infection for animals that began treatment on day 4 post infection. Negative values indicated weight loss. **(E)** Average body temperatures for animals that began treatment on day 4 post infection. **(F)** Average clinical scores for animals that began treatment on day 4 post infection. **(G)** Pictures of primary lesions (necrosis only) from representative animals at 7 dpi. Bars represent 1cm. Black circles denote site of intradermal inoculation.

**Figure 2:**
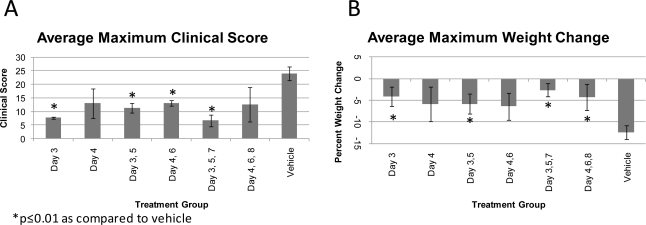
Disease severity measurements for evaluation of 1, 2 or 3 doses of CMX001 given every other day beginning at day 3 or 4 post infection. Animals were dosed at concentrations and schedules as outlined in Table 1. **(A)** Average of maximum clinical scores for each animal per group over the course of the experiment. **(B)** Average of maximum percent weight loss from weight at day 0 for each animal per group over the course of the experiment. *p≤0.01 as compared to vehicle

**Figure 3:**
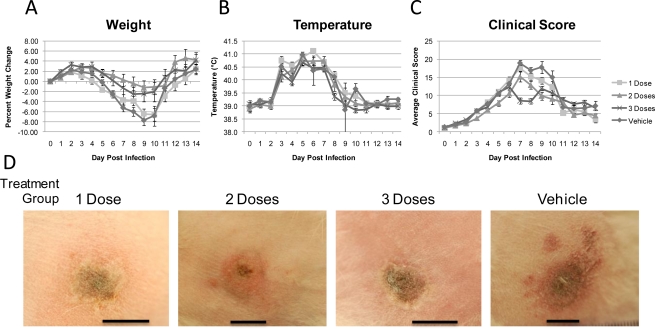
Animals treated beginning at the appearance of secondary lesions. Groups of 12 animals were treated with CMX001 1, 2 or 3 times every other day beginning at the appearance of secondary lesions in the ears. Animals were dosed at concentrations and schedules as outlined in Table 2. **(A)** Average weight change over time for each group. Negative values represent weight loss. **(B)** Average body temperatures over time. Temperatures over 39.5 °C are considered a fever. **(C)** Average clinical score over time. Error bars represent SEM. **(D)** Photographs of primary lesions from each treatment group at 8 dpi. Bar represents 1cm.

**Figure 4:**
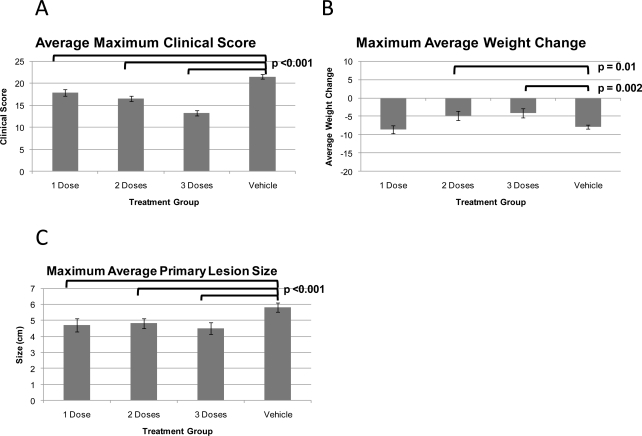
Disease severity measurements when CMX001 treatment began at the appearance of secondary lesions. Animals were dosed at concentrations and schedules as outlined in Table 2. **(A)** Average of maximum clinical scores for each animal per group over the course of the experiment. **(B)** Average of maximum percent weight loss from weight at day 0 for each animal per group over the course of the experiment. **(C)** Average of maximum diameters of primary lesions of each animal per group over the course of the experiment.

**Figure 5:**
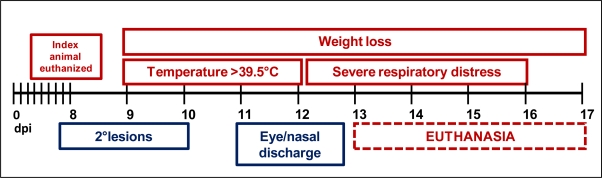
Time line of disease in 8–10 week old New Zealand White rabbits infected via natural aerosol. Symptoms in red boxes represent clinical measurements that contribute to euthanasia guidelines. Clinical symptoms are indicated in blue boxes. Reproduced with permission from American Society for Microbiology [12].

**Figure 6:**
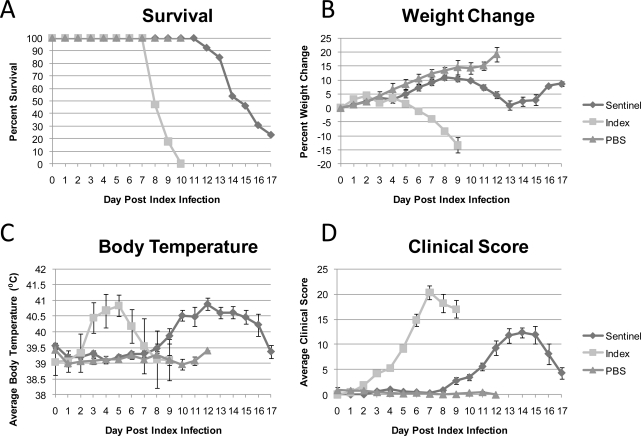
Disease in rabbits lethally infected with RPV via natural aerosol. **(A)** Survival of naturally infected rabbits (sentinel), infected cage mates providing aerosolized RPV (index) and uninfected control animals (PBS). **(B)** Average weight change from weight at time of infection for each group. Negative values represent weight loss. **(C)** Average body temperatures over time. Temperatures over 39.5 °C are considered a fever. **(D)** Average clinical score over time. Error bars represent SEM. Reproduced with permission from American Society for Microbiology [12].

**Figure 7:**
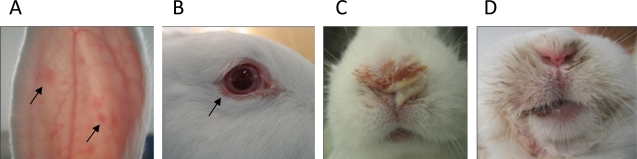
Presentation of disease in rabbits infected by aerosol transmitted RPV. Rabbits were infected as described in Figure 5. **(A)** Secondary lesions observed in the ears of animals exposed to RPV via aerosol. Arrows show locations of 2 representative lesions in the ear. **(B)** Photograph of a secondary lesion on the eye of an animal marked with arrow. **(C)** Normal profile of intradermally lethally infected RPV rabbit nose. **(D)** Profile of aerosol infected RPV rabbit showing severe swelling of nose and muzzle. Reproduced with permission from American Society for Microbiology [12].

**Figure 8:**
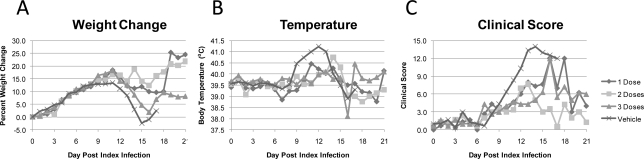
Aerosol infected animals treated beginning at the appearance of secondary lesions. Groups of 3 animals were treated with CMX001 1, 2 or 3 times every other day beginning at the appearance of secondary lesions in the ears. Animals were dosed at concentrations and schedules as outlined in Table 3. **(A)** Average weight change over time for each group. Negative values represent weight loss. **(B)** Average body temperatures over time. Temperatures over 39.5 °C are considered a fever. **(C)** Average clinical score over time.

**Table 1:** Evaluation of 1, 2 or 3 doses of CMX001 given every other day beginning at day 3 or 4 post infection. CMX001 was administered once a day (QD).

**CMX001 Dose (mg/kg)**	**Day of Dosing (dpi)**	**Mean Time to Death ± SEM**	**Survival at Day 14PI**
20	3	NA	3/3 (100%)*
20	4	10 ± 0	2/3 (66%)
20	3, 5	NA	3/3 (100%)*
20	4, 6	7 ± 0	2/3 (66%)
20	3, 5, 7	NA	3/3 (100%)*
20	4, 6, 8	9 ± 0	2/3 (66%)
Vehicle	Vehicle	9 ± 0	0/2 (0%)

* p = 0.099 as compared to vehicle by unpaired t-test.

**Table 2:** Evaluation of efficacy of CMX001 when treatment is begun at the appearance of secondary lesions in the ears. Groups of 12 animals were treated with CMX001 1, 2 or 3 times every other day beginning at the appearance of secondary lesions in the ears.

**CMX001 Dose (mg/kg)**	**Frequency of Dosing**	**Day Dosing Began (dpi)**	**Mean Time to Death ± SEM**	**Survival at Day 14PI**
20	1 dose	3 to 4	10.6 ± 0.24	7/12 (58.33%)
20	2 doses	3 to 5	10.5 ± 0.96	8/12 (67.67%)
20	3 doses	3 to 4	11 ± 0	11/12 (91.67%)
Vehicle	NA	3 to 4	9.06 ± 0.18	4/36 (11.1%)

**Table 3:** Evaluation of efficacy in natural aerosol infected animals. Groups of 3 animals were treated with CMX001 1, 2 or 3 times every other day beginning at the appearance of secondary lesions in the ears.

**CMX001 Dose (mg/kg)**	**Frequency of Dosing**	**Day Dosing Began (dpi)**	**Mean Time to Death ± SEM**	**Survival at Day 14PI**
20	1 dose	7 to 8	18 ± 0	2/3 (66%)
20	2 doses	7 to 9	13 ± 0	2/3 (66%)
20	3 doses	7 to 9	16 ± 0	2/3 (66%)
Vehicle	NA	NA	14.3 ± 1.3	0/3 (0%)
